# Reviews

**Published:** 1886-07

**Authors:** 


### REVIEWS.

First Annual Report of the State Board of Health, of the State of Maine, for the year ending December 31st, 1885.

Augusta, 1886.

The act to establish the above Board, was approved on February 27th, 1885, and the following members appointed: Frederick H. Gerrish, M.D., President; A. G. Young, M.D., Secretary; Hon. Lewis Barker, Hon. Stephen J. Young, O. A. Horr, M.D., E. C. Jordan, C.E., J. O. Webster, M.D.

The first meeting was held April 13th, of the same year. Six meetings have already been held, and the amount and character of the work performed, as shown by the Report, speak well for the intelligence and energy of the Board, and promises for the State an improved sanitary condition for the future.

Circulars relating to the restriction and prevention of contagious diseases have been published and circulated extensively, especially on cholera, typhoid fever, diphtheria, scarlet fever and small pox. Also on the building and ventilation of school-houses.

A full account of the small pox epidemic in Montreal, is given, and the protective measures there adopted.

A very important and interesting work established by the Board, was the sending out of letters of inquiry to all the physicians in the State, regarding the topography and epidemiological conditions of their localities. The results, as is shown by the reports from their correspondents, proved of great value. Interesting accounts from the older members of the medical profession, of the prevalence of cholera, yellow fever, etc., will be found amongst them.

There seems to be considerable difference of opinion, in regard to the contagiousness of diphtheria. On this point, Dr. Morton, of Bethel, gives testimony in its favor. He says: "I think that sufficient notice is not taken of the part that *cats* and *dogs* play, in the spread of this disease. These animals have free access to children sick with the above disease, and they prowl for miles from home, carrying the poison germs in their hair, and in their plays and fights communicate it to other animals, who convey it into their families, and are carressed and handled by children, and they become victims to those diseases, and people are at a loss to know how they became thus affected."

An Augusta physician relates that about a year ago the pet kitten of one of his children became sick with what appeared like diphtheria. There was no other case, nor had been lately in town.

Apropos of this subject, Dr. Jacobi gives an account of a diphtheria epidemic in a hen coop. "Twenty-six hundred hens were imported from Verona, Italy, into a village, Messelhausen, in Baden, Some of these hens were affected with diphtheria when they arrived. Within six weeks six-hundred of their number died of diphtheria, and eight hundred more soon after. In the following summer, one thousand chickens were raised by artificial breeding, all of which died of diphtheria, within six weeks. Five cats kept in the place also died of diphtheria ; a parrot fell sick with it, but recovered. Besides, four of the six working men, employed in taking care of the hens of the establishment, were taken sick with diphtheria. Not a single case, however, occurred in the neighboring village. Thus, it is safe to assume that the diphtheritic disease of hens can be transmitted to man."

As regards the contagiousness of consumption, the correspondents, in a large proportion, have decided convictions in its favor. The opinions of the Veterinarians tend to the same belief. By way of prophylaxis banishment to hospitals is condemned as cruel and unnecessary. Destruction of the sputa, general cleanliness and proper ventilation are the means suggested. The patient should always sleep alone. Under these circumstances the danger of contagion is slight.

Several special papers of interest, relating to sanitary matters, are to be found at the end of the Report. Dr. Gerrish, the President, gives a condensed account of the work accomplished by the the American Public Health Association. Mr. Jordan shows how death rates are affected by defective plumbing and sewerage, and Dr. Horr tells all there is to be known on vaccination. The system of local Boards in towns and cities, and their importance, is from the pen of Dr. Webster.

A glossary is appended so that the general public will find no stumbling blocks in the shape of unknown technical terms.

On the whole, the Report is the most complete we have yet seen, and we recommend its careful perusal to those in other States, who have in view the formation of similar institutions. J. O. L.

Osservazioni Anatomische, del. Prof. Lombardini. Sull 'utero di cavalla nei primi mesi della gravidanza.

In this pamphlet the author gives some exceedingly interesting descriptions of the anatomy of the uterus of the horse, during the first months of pregnancy. It contains five illustrations, showing the histology of the corion placenta, etc. The observations made thereon, will be found of great value to the Veterinarian who makes a special study of obstetrics.

In relation to the bare spaces of the placenta, which Turner has found in the whale, Eschricht in the common seal, and Horne in the Malayan tapir, he says that they are not mentioned by those who have studied the equine uterus, and relates that in 1880 he had occasion to examine the uterus of a horse, in the seventh month of pregnancy. To the naked eye the crypts appeared to have been arrested in their circumference, forming a clean border. On microscopical examination, however, it became manifest that they were portions of the mucous membrane, in which the placental follicles were incompletely developed.

Outlines for a Museum of Anatomy, prepared for the Bureau of Education. by Dr. R. W. Shufeldt, U.S.A., Washington, 1885.

This pamphlet is well worth reading by any one interested in the subject of which it treats, but especially so by the veterinarian and the student of comparative medicine.

Dr. Shufelt starts out with a general condemnation of the few anatomical museums in this country, as they are now conducted. There is no system, he says, in the arrangement, and the specimens are almost useless. He considers vain, all the labor expended on dried, dissected preparations.

He gives an idea of what a model museum should be, and in stating the laws which should govern its formation, he reiterates a truth, often expressed in the pages of this Journal, viz.: that *progress in medicine and surgery is in proportion to the advance made in the knowledge of the Anatomy, Physiology and Pathology of the lower animals*

He thinks that the law of evolution should be kept in view in the arrangement of the museum, and that the classification of its specimens should tend to its illustration. Each specimen should occupy its proper place, and should not be considered merely as an object of curiosity.

Classification of the forms of the animal kingdom are explained, methods are suggested for the exhibition of anatomical subjects, and rules laid down to best enable the student to study the grand principles on which morphology, the "back-story" of medicine is founded.

Educational Museums of Vertebrates. An address before the Section of Biology, of the A. A. A. S., at Ann Arbor, August 1885. By Burt G. Wilder, M. D., Vice-President.

"Order is Heaven's first law," and Dr. Wilder believes that this law should prevail in terrestrial as well as celestial affairs, especially in the arrangement of museums of Vertebrate animals, one of which should be found in every educational institute.

He thinks an institute of this character should not only exhibit *facts*, but also illustrate *ideas*, and explains the methods employed in the collection at Cornell University, the specimens in which are arranged in harmony with the law of evolution. Such work, in his opinion, would be a more appropriate memorial of the immortal Darwin, than the statue which had been recently dedicated to him in London.

Glover's Album. A treatise on canine diseases. By H. Clay Glover, V.S.

Of convenient size to carry in the pocket, this little book will prove of great value to owners of dogs.

The diseases common to these animals are described, and the treatment explained, while some excellent formula are added. The portraits of the most prominent dogs are also to be found in it. We recommend it to all who would keep their canine friends in good condition.

Thirteenth Annual Report of the New Jersey State Board of Agriculture, 1885.

In this volume is to be found the reports of the State Veterinary Inspectors, in which inoculation of cattle suffering from pleuro-pneumonia, is found of great value as a preventive measure.

It demonstrates that immediate destruction of the affected animal, is the only remedy in cases of glanders. Attention is called to necessity of more stringent laws to regulate the meat and milk traffic of cows affected with tuberculosis. Dr. Wm. H. Lowe, in his report, says: "Pleuro-pneumonia principally involves a question of property and its money value, but tuberculosis, that is, if it be communicable to the human subject, through the milk and meat of tuberculosis animals, as many eminent scientists now claim, has a significance of vast and wide-spread importance. As the law now stands, there is no authority by which tuberculos animals can be destroyed, and yet, considering the prevalence of the disease, and its devastation, it is a question whether any of the other contagious diseases are worthy of more attention, or stricter legislation than tuberculosis."

The same inspector reports a case of actinomycosis, and also gives an account of a strange case in Passaic city A number of cows became sick, and the disease was variously pronounced as meningitis, enteritis, &c. Dr. Lowe diagnosed lead poisoning, and his opinion was justified by the finding of a number of paint pots and brushes in the pasture grounds. In regard to rabies, he thinks that whatever may be the results of Pasteur's investigations the importance of prevention cannot be underated.

